# Genetic Relationship between *Salmonella* Isolates Recovered from Calves and Broilers Chickens in Kafr El-Sheikh City Using ERIC PCR

**DOI:** 10.3390/ani12233428

**Published:** 2022-12-05

**Authors:** Rasha Gomaa Tawfik, Mahmoud F. Gawish, Mahmoud M. Abotaleb, Hassan S. Nada, Kareem Morsy, Mohamed M. A. Abumandour, Helmy Torky

**Affiliations:** 1 Department of Microbiology, Faculty of Veterinary Medicine, Alexandria University, Alexandria 21523, Egypt; 2 Department of Bacteriology, Mycology and Immunology, Faculty of Veterinary Medicine, Kafr-Elsheikh University, Kafr-Elsheikh 33516, Egypt; 3 Central Laboratory for Evaluation of Veterinary Biologics, Agriculture Research Center, Cairo 11381, Egypt; 4 Biology Department, College of Science, King Khalid University, Abha 62529, Saudi Arabia; 5 Zoology Department, Faculty of Science, Cairo University, Cairo 12613, Egypt; 6 Department of Anatomy and Embryology, Faculty of Veterinary Medicine, Alexandria University, Alexandria 21523, Egypt

**Keywords:** *Salmonella*, broilers, calf, antimicrobial resistance, β-lactamases, integrons, ERIC PCR, zoonosis

## Abstract

**Simple Summary:**

Our study was designed for the *Salmonella* diagnosis from chickens and calves, to determine its resistance to antimicrobials’ phenotypic and genotypic characterization of integrons and β lactamase genes in the multidrug resistance of different *Salmonella* serotypes, and to detect the genetic relationship between isolated Salmonella using ERIC PCR. Isolated poultry Salmonella were identified as *S. Typhimurium*, *S. Enteritidis*, and *S. Kentucky*, but isolated cattle Salmonella were *S. Enteritidis* and *S. Kentucky*. An RT PCR was applied for identifying the accompanying class 1 integrons and ESBLs from MDR *Salmonella* isolates (two isolates of *S. Kentucky* were divided as one from calf and one from poultry). Our results detected a *blaTEM* and class 1 *integron*, but were negative for *bla IMP*, *bla VIM*, and *bla SHV*. An ERIC PCR was conducted for understanding the clonal relationship among various β-lactamase-producing MDR *Salmonella* isolates. Two isolates of *S. Enteritidis* isolated from poultry and calves had 100% similarity, despite indicating that there were interactions between broilers and calves living on the same farm that caused infection from the same *Salmonella* strains, while the other two isolates of *S. Kentucky* showed only 33% serovarities.

**Abstract:**

A prevalent bacterial intestinal infection with severe economic damage is salmonellosis. Our study was carried out to diagnose *Salmonella* from chickens and calves, to determine its resistance to antimicrobials’ phenotypic and genotypic characterization of integrons and β lactamase genes in the multidrug resistance of different *Salmonella* serotypes, and to detect the genetic relationship between *Salmonella* isolates collected from different origins using an ERIC PCR. In total, 200 samples from diseased chicken and diarrheic calves were obtained from 50 various farms from Kafr El-sheikh, Egypt. *Salmonella* poultry isolates were characterized as *S. Typhimurium* (3/8), *S. Enteritidis* (3/8), and *S. Kentucky* (2/8), but *Salmonella* isolates from cattle were *S. Enteritidis* (1/2) and *S. Kentucky* (1/2). When antibiotic susceptibility testing was completed on all of the isolates, it showed that there was multidrug resistance present (MDR). A PCR was applied for identifying the accompanying class 1 integrons and ESBLs from MDR *Salmonella* isolates (two isolates of *S. Kentucky* were divided as one from calf and one from poultry). Our results detected *blaTEM* and class 1 *integron*, but were negative for *bla IMP*, *bla VIM*, and *bla SHV.* An ERIC PCR was conducted for understanding the clonal relation between various β-lactamase-producing MDR *Salmonella* isolates. The same four previously mentioned isolates were also tested. The two isolates of *S. Enteritidis* isolated from poultry and calves had 100% similarity despite indicating that there were interactions between broilers and calves living on the same farm that caused infection from the same *Salmonella* strains, while the other two isolates of *S. Kentucky* showed only 33% serovarities.

## 1. Introduction

A frequent bacterial intestinal infection caused by Salmonella serovars is salmonellosis [1]. It affects both humans and the majority of animals, which facilitates transmission and cross-contamination [2]. Enterobacteriaceae, a family of rod-shaped, Gram-negative bacteria, includes Salmonella. Clinical manifestations can include people with acute or chronic enteritis or septicemia as well as healthy chronic carriers. Poultry has been recognized as one of the main sources for disseminating *Salmonella* infection to man and animals [3]. *Salmonella* species can cause a wide range of clinical signs in calves, including diarrhea and possible dysentery, joint infections, chronic pneumonia, and sudden death from septicaemia. Direct contact with cattle and/or eating foods containing cow’s milk can both cause salmonellosis in humans (milk and uncooked beef meat) [4]. However, the isolation of *S. Typhimurium* and *S. Enteridis* from calves and poultry in the same area is probably indicative of fecal contamination at the source. *Salmonella* can persist for several months in the farm environment, including in water [5]. Serotyping, which offers details about the severity of the illness, the source of contamination, and the resistance pattern, is a crucial tool for understanding the epidemiology of Salmonella infections [6]. 

The widespread issue of multidrug resistance (MDR), which is encoded by resistance genes on integrons, is a serious one. Mobile DNA fragments known as integrons play a crucial part in the transmission of antibiotic resistance genes in Gram-negative bacteria [7]. The site-specific recombinase responsible for cassette insertion is encoded by the inte-grase gene (intI1), which is present in the majority of the class 1 integrons seen in clinical isolates [8,9]. The attI1 site on the integrase gene is where the cassettes are integrated, and Pc is the promoter that triggers the transcription of the cassette-encoded genes [8,10]. Whereas a number of the most recently discovered ESBL genes are typically figured out within integron-like structures including blaTEM or blaSHV, ESBL-encoding genes are typically figured out on conjugative plasmids (such as blaTEM or blaSHV) (blaCTX-M, blaGES, or blaVEB-1) [11,12]. Contrarily, isolates that produce ESBLs frequently exhibit resistant to certain other antibiotics, such as aminoglycosides, tetracyclines, chloramphenicol, trimethoprim, sulfonamides, or quinolones. A genetic structure created by the synergistic generation of diverse interactions includes different resistance genes on plasmids, transposons, or integrons as transferable elements or genetic structures [12,13]. The dissemination of these genetic elements can be aided by the existence of ESBL genes on integrons [11,14].

Acknowledging the clonal relationship between different lactamase-producing MDR Salmonella isolates using the enterobacteria repetitive intergenic consensus (ERIC) polymerase chain reaction (PCR) strain is crucial for both identifying the source of infection and preventing the spread of nosocomial pathogens. The cross-infection between animal and poultry species by *Salmonella* may be accrued through the ingestion of contaminated water and food [15]. Therefore, our investigation focused on the isolation and identification of *Salmonella* serovars from diseased chicken and diarrheic calves and declaring associated antimicrobial resistance profiles: the characterization of β-lactamase-encoding genes (*blaTEM*, *bla IMP*, *bla VIM*, and *bla SHV*), and class 1 *integron* (int) in addition to identifying the genetic relation among the same *Salmonella* serovar but from various origins using (ERIC PCR).

## 2. Materials and Methods

### 2.1. Samples Collection

Two hundred samples (one hundred fecal and rectal swabs from diarrheic calves aging up to 3 months, and one hundred samples from freshly dead broiler chickens suspected to be infected with *Salmonella*) were transported immediately in an ice box to the lab for bacteriological examination for *Salmonella* (Table 1). They were enriched in peptone water at 37 °C at 24 h for non-selective pre-enrichment then transferred onto 10 mL of Rappaport–Vassiliadis broth for selective enrichment. This broth was incubated aerobically at 42 °C for 18 h. A loopful from the culture was streaked into the surface of MacConkey agar for 24 h at 37 °C which was selective for Enterobacteriaceae. Colorless colonies on MacConkey agar were inoculated into the Xylose lysine deoxycholate (X.L.D.) surface and *Salmonella-Shigella* agar (S.S) media for selective plating. The inoculated plates were incubated at 37 °C for 24–48 h. Suspected colonies were characterized morphologically according to [16]. These colonies were picked up and sub-cultured, and a loopful of each pure culture was inoculated into the nutrient slope and semisolid agar tube for further biochemical and serological identification.

### 2.2. Biochemical Identification

It was done in accordance with [16,17,18,19]. All suspected Salmonella colonies were selected from the agar plates and inoculated onto the following biochemical test tubes for confirmation: the triple sugar iron (TSI) test (suspicious Salmonella colonies are thought to produce colonies that are black or have black centers and red medium on TSI agar; OXOID, Hampshire, UK), the citrate test (suspicious Salmonella colonies are thought to produce colonies that are blue in color for the citrate test), and the urease test (sus at 37 °C, plates were incubated for 24 or 48 h).

### 2.3. Serological Identification 

A total of 10 *Salmonella* isolates (2 isolates from poultry and 8 from calves) were identified morphologically and biochemically as *Salmonella* were subjected to serological identification according to the Kauffman-white scheme [20] using the slide agglutination technique with the polyvalent somatic (O) and flagellar (H) antisera (Welcome Diagnostic, Birmingham, UK).

### 2.4. Antimicrobial Sensitivity Testing (Phenotypic Resistance)

The susceptibility of *Salmonella* isolates to different antimicrobial agents was discovered by the Kirby–Bauer method (Disc diffusion test) based on the criteria of the Clinical and Laboratory Standards Institute (CLSI, 2011). A total of 14 antimicrobials were chosen, according to their common usage for treatment and prevention of the *Salmonella* infection in poultry and humans, and they were used for sensitivity tests of 10 isolated S. enterica. The antimicrobial discs included ampicillin (AMP 10 Mg), Ciprofloxacin (CIP 5 Mg), cefotaxime (CTX 30 Mg), Chloramphenicol (C 30 Mg), Streptomycin (STR 10 Mg), nalidixic acid (NAL 30 Mg), trimethoprim-Sulfamethoxazole (SXT 25 Mg), and tetracycline (TE 30 Mg), Enrofloxacin (ENR 5 Mg), Vancomycin (VA 30 Mg), Kanamycin (KAN 30 Mg), ceftriaxone (CRO 30 Mg), Erthyomycin (E 15 Mg), and Amoxicillin (AMC 30 Mg).

### 2.5. Molecular Identification of B-Lactamase Encoding Genes, Class 1 Integron, and ERIC PCR of Isolated Salmonella 

We modified the manufacturer’s instructions for the QIAamp DNA Mini kit (Qiagen, Hilden, Germany, GmbH) to extract DNA from the samples (Table 2). Briefly, three to five representative colonies of the same morphological type were taken from the slants of the previously isolated bacteria and enriched into a tube containing 2 mL of tryptic soya broth (TSB) for 18 h at 37 °C. Then, 200 µL of the sample suspension was incubated with 10 µL of proteinase K and 200 µL of lysis buffer at 56 °C for 10 min. After incubation, 200 µL of 100% ethanol was added to the lysate. Then, the sample was washed and centrifuged according to the manufacturer’s recommendations. Nucleic acid was eluted with 100 µL of elution buffer provided in the kit. 

PCR reaction primers were used in a 25 µL reaction and had 12.5 µL of EmeraldAmp Max PCR Master Mix (Takara, Shiga, Japan), 1 µL of each primer of 20 pmol concentrations, 5.5 µL of water, and 5 µL of the DNA template. The reaction was performed in an applied biosystem 2720 thermal cycler. The products of PCR were separated by electrophoresis on 1.5% agarose gel (Applichem GmbH, Darmstadt, Germany) in 1x TBE buffer at room temperature using gradients of 5V/cm. For the gel analysis, 20 µL of the multiplex PCR products were loaded in each gel slot. Gelpilot 100 bp DNA ladder (Qiagen GmbH, Hilden, Germany) and generuler 100 bp ladder (Fermentas, Thermo, Hamburg, Germany) were used to determine the fragment sizes. The gel was photographed by a gel documentation system (Alpha Innotech, Biometra, San Leandro, CA, USA), and the data were analyzed by computer software. ERIC fingerprinting data were transformed into a binary code depending on the presence or absence of each band. Dendrograms were generated by the unweighted pair group method with arithmetic average (UPGMA) and Ward’s hierarchical clustering routine. A similarity index (Jaccard/Tanimoto Coefficient and the number of intersecting elements) between all samples was calculated using the online tool (https://planetcalc.com/1664/, accessed on 21 May 2022).

## 3. Results

### 3.1. The Prevalence of Salmonella Isolated from Different Spp.

In this study, Salmonella enterica isolates were obtained from chickens and calves representing commonly isolated serotypes in Egypt. The overall incidence of *Salmonella*, in this investigation, was 2% in the chickens’ samples and 8% in the calves’ samples (Table 3).

### 3.2. Serological Identification of Salmonella Isolates

Concerning serotyping, there were three *Salmonella* isolates identified which included *S. Kentucky*, *S. Typhimurium*, and *S. Enteritidis* from all isolates as shown in Table 4. Three *Salmonella* serovars were identified from poultry as (*S. Kentucky* (2/8), *S.Typhimurium* (3/8), and *S. Enteritidis* (3/8)), while in cattle there were two serovars identified serologically, *S. Kentucky* (1/2) and *S. Enteritidis* (1/2) as shown in Table 5.

### 3.3. The Antimicrobial Resistance

As shown in Table 6, the highest antibiotic resistances belonged to Macrolides antibiotics Erythromycin 100%, Vancomycin 90%, B-lactamase penicillin, Ampicillin 90%, Amoxicillin 80%, and quinolones nalidixic acid 90%, despite, the fact that it showed variable resistance to Aminoglycosides antibiotics Streptomycin 60%, Kanamycin 40%, Sulphonamides, antibiotics trimethoprim-Sulfamethoxazole 40%, and tetracycline 40%. *Salmonella* isolates showed sensitivity to cephalosporin ceftriaxone 60%, and Phenicols antibiotic Chloramphenicol 60%. In this study, most of the *Salmonella* isolates showed multidrug-resistance of 80% to ≥5 of the fourteen antimicrobials used.

### 3.4. Prevalence of β-Lactamase-Encoding Genes in Salmonella Isolation

**Figure 1:**
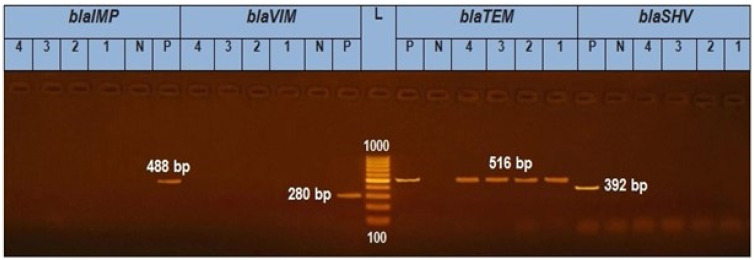
In total, 1.5% Agarose gel electrophoresis for PCR results of blaTEM gene in *Salmonella* isolates (516 bp), blaVIM (280 bp), blaIMP (488), and blaSHV (392 bp). L is a 1000 bp ladder used as a size marker. P is controlled positively. N is controlled negatively.

A uniplex PCR was applied for the discovery of bla TEM, bla SHV, bla VIM, and bla IMP. It was exhibited that the four isolates (two *S. Enteritidis* and two *S. Kentucky*) tested genetically have obtained bla TEM amplicon (516 bp) with a percentage of 100%. As shown in (Figure 1), isolates 1, 2, 3, and 4 were positive for the blaTEM gene (516 bp) but negative for the blaSHV gene (392 bp) and blaVIM gene (280 bp).

### 3.5. Prevalence of Class 1 Integrons for MDR Salmonella

**Figure 2:**
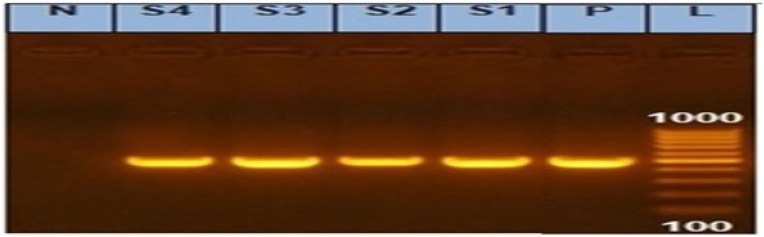
Agarose gel showing amplified products of integrase gene (491 bp) in *Salmonella* isolates. N: Control Negative; P: Control Positive; L: DNA ladder 100 bp; and Lanes (S1, S2, S3, and S4) examined *Salmonella* isolates (S1: *S. Kentucky* from calf and S2: *S. Kentucky* from chicken) and (S3: *S. Entertidis* from chicken and S4: *S. Enteritidis* from the calf).

**Figure 3:**
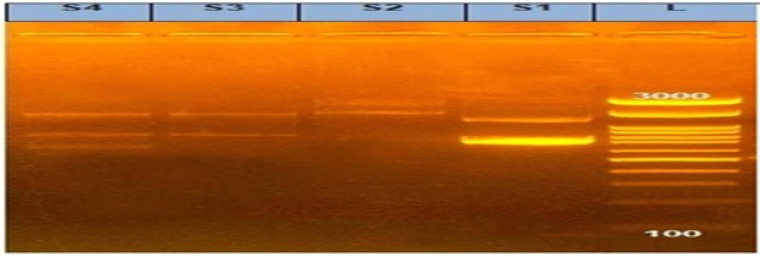
Agarose gel showing amplified products of variable integrons in *Salmonella* isolates. L: DNA ladder 100 bp and Lanes (S1, S2, S3, and S4) examined *Salmonella* isolates (S1: *S. Kentucky* from calf and S2: *S. Kentucky* from chicken) and (S3: *S. Entertidis* from chicken and S4: *S. Enteritidis* from the calf).

In our investigation, the PCR screening results detected amplified products of int1 amplicon (491 bp) with a percentage of 100%, as shown in Figure 2 and Figure 3.

### 3.6. ERIC PCR 

**Figure 4:**
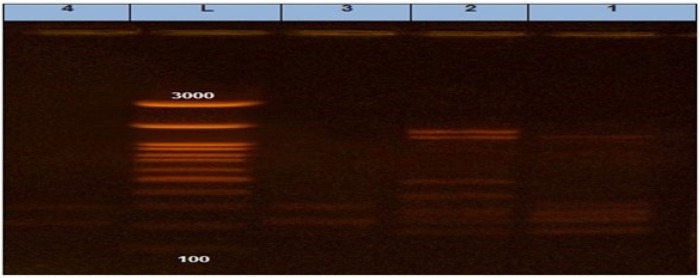
ERIC PCR showing different bands in each isolate.

**Figure 5:**
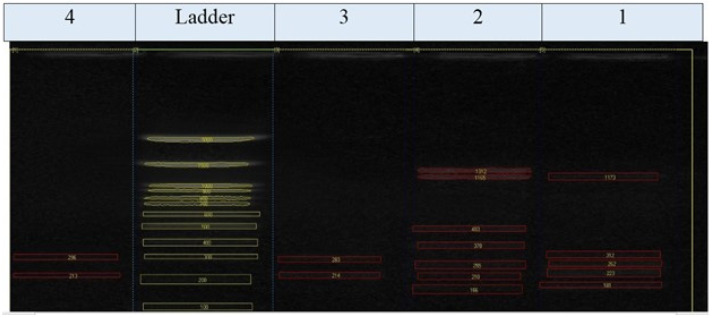
ERIC bands (measured by BioDocAnalyze software for installation and image acquisition on the computer).

**Figure 6:**
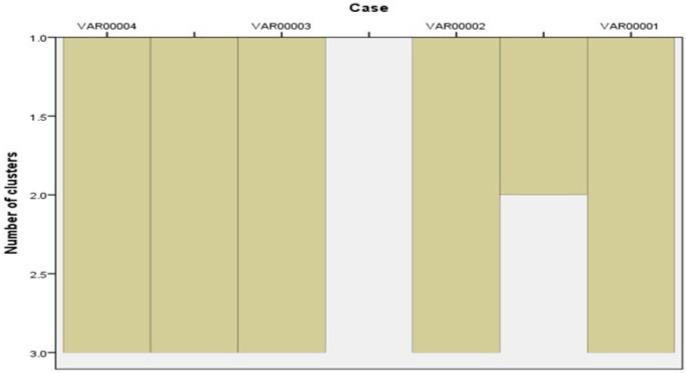
Dendrograms were generated by the unweighted pair group method with arithmetic average (UPGMA) and Ward’s hierarchical clustering routine. Similarity index (Jaccard/Tanimoto coefficient and the number of intersecting elements) between all samples were calculated using the online tool (https://planetcalc.com/1664/, accessed on 21 May 2022).

**Figure 7:**
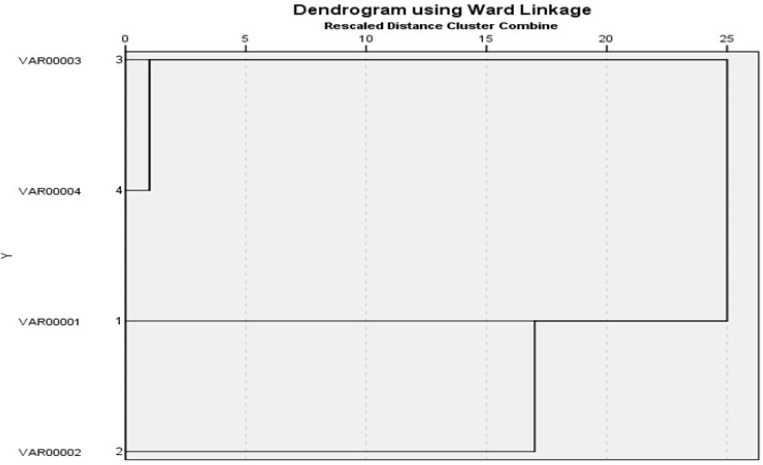
Dendrograms were generated by the unweighted pair group method with arithmetic average (UPGMA) and Ward’s hierarchical clustering routine. Similarity index (Jaccard/Tanimoto coefficient and the number of intersecting elements) between all samples were calculated using the online tool (https://planetcalc.com/1664/, accessed on 21 May 2022).

It was found that two *S. Enteritidis* isolates (one from cattle and the other one from chicken) had 100% similarity according to (Jaccard/Tanimoto coefficient and the number of intersecting elements), despite the fact that two *S. Kentucky* isolates (one from cattle and the other one from chicken) had 33% similarities as shown in Table 7 and Figure 4 and Figure 5. Dendrograms were produced using 234 Ward’s hierarchical clustering procedure and the unweighted pair group technique with arithmetic average (UPGMA), as shown in Table 8 and Figure 6 and Figure 7.

## 4. Discussion

One of the most widely prevalent causes of both human and animal food-borne diarrheal illness is Salmonella [27]. The majority of the 2500 Salmonella serovars have a wide range of hosts and can infect a variety of hosts [28]. According to the nation, the type of manufacturing method, and the particular control mechanisms in place, the prevalence of Salmonella serovars in samples can change [29]. In the present study, 8 out 100 chicken samples were positive for *S. Enterica* representing (8%); this result is in good harmony with a study applied by [30,31], who reported (8.6% and 8%), respectively, in the chicken farms. Three serovars were identified in chicken samples which included *S. Kentucky* 25% (2/8), which was close to [32,33], and *S. Typhimurium* 37.5% (3/8) and *S. Enteritidis* 37.5% (3/8), which were quite similar to [33,34]. The prevalence of *Salmonella* from 100 fecal calves’ samples was 2% (2 isolates), which was close to that obtained from (1.5%) [35]. Subsequently, the isolation percentage among the diseased calves was lower than in chicken, which agreed with [29].

Concerning the antibiotic sensitivity test, the highest antibiotic-resistant *Salmonella* isolates were Erythromycin 100%, Vancomycin 90%, Ampicillin 90%, Amoxicillin 80%, and nalidixic acid 90%, which agree with the previous study applied by [36,37]. In this study, most of the *Salmonella* isolates showed multidrug-resistance of 80% to ≥5 of the fourteen antimicrobials used, and the obtained results matched with the results reported by [36,38,39,40]. They were also similar to [41], which reported that numerous *Salm.* Typhi, *Salm.* Paratyphi A, *Salm.* Typhimurium, and *Salm.* Enteritidis isolates were MDR as they had a resistance to a minimum of four antibiotics. Salmonella typhimurium, Salmonella enteritidis, Salmonella typhi, and Salmonella paratyphi A isolates were all discovered to be susceptible to ciprofloxacin and ofloxacin. 

The spread of antibiotic resistance 262 in Salmonella enterica species is greatly aided by the horizontal transmission of resistance genes. These resistance genes can be located on chromosome 263 of bacteria or on resistant plasmids. Generally, the most effective way to transfer resistance is by the horizontal transmission of genes via plasmids, which is happening frequently and involves multiple resistance genes at once [42]. Transposons, integrons, and plasmids that gain resistance genes have the potential to spread to various strains or species. Transposons, considered a genetic element, move about and have the ability to carry resistance genes. They also have the ability to recombine resistance genes with plasmids or chromosomes thanks to their transposase activity. Integrons are made up of a promoter, a recombination site that integrase can recognize, and the recombination enzyme integrase (encoded by the intI gene). Moreover, these components are required for the expression of the gene cassettes found within an integron [43]. These configurations, particularly on plasmids, effectively encourage the acquisition of external genes such as genes for antibiotic resistance in the bacterial genome. Additionally, conjugation events make it easier for resistance genes to move from plasmids to other strains or species via transposon or integrin [42]. Most Enterobacteriaceae produce extended spectrum-lactamases (ESBLs), which is a key mechanism for granting resistance. According to the substrate and inhibitor mechanisms, there are various types of ESBLs [44]. The first lactamase discovered was TEM-1, which was obtained from an *E. coli* strain that belonged to a patient in Greece by the name of Temoniera [45]. TEM-3 [44] was the first member of the TEM-type lactamase family to exhibit ESBL properties [44]. *Salmonella* spp. has been found to have TEM-type lactamases [45,46,47]. Another lactamase is SHV (sulphydryl variable), which is typically seen in *Klebsiella pneumoniae* and *E. coli* [45]. There are more than 90 different TEM, and more than 25 different SHV varieties of lactamases, which are the most prevalent and widely dispersed in nature [45,47]. SHV is plasmid-encoded. With more than 90 varieties of TEM and more than 25 types of SHV, the TEM and SHV types of lactamases are the most prevalent and extensively distributed in nature [46,48].

In this study, a PCR was performed on two *S. Kentucky* isolates and two *S. Enteritidis* isolates of various origins to figure out the extended spectrum β-lactamases genes (*bla TEM*, *bla SHV*, *bla VIM*, *bla IMP*), and finally class 1 *integron* encoding genes. The obtained results revealed the detection of *blaTEM* and class 1 *integron*, but were negative for *bla IMP*, *bla VIM*, and *bla SHV*. These results agreed with the work by Zhao and Moawad [49,50], and this result is similar to the result obtained by Bahatta et al. [41], which reported that research using PCR amplification and sequencing on all Salm. Enteritidis isolates with resistance illustrated the existence of the ESBL gene blaSHV; however, the genes blaTEM and blaCTX were missed. 

Several methods exist for investigating the molecular diversity of bacteria. These strains’ random entire genomes were typed using the RAPD and ERIC PCR techniques. With a discriminating value (DI) of 0.9821 and 100% repeatability, ERIC PCR was discovered to be extremely effective. According to Nath et al. [51], RAPD had poor repeatability (40%) but was effective in differentiating the strains (DI = 0.8978). Regular epidemiological research can benefit from using ERIC PCR because of its shown discriminating power and ease of usage.

Salmonella and other pathogenic members of the Enterobacteriaceae family are studied genetically using the ERIC PCR method. This method depends on the creation of complex and repeatable fingerprints by amplifying a genomic DNA fragment using a single primer pair that is complementary to short repeat sequences. The same four previous mentioned isolates were also tested using the ERIC PCR. Another interesting finding was that two isolates of *S.Enteritidis* originating from poultry and calves had 100% similarity, despite the other two isolates of *S. Kentucky* showing only 33% serovarities. The current findings came in contact with those illustrated by Secundo de Souza et al. [52] which reported that some of the SG isolates improved from the various areas at certain times clustered with 100% similarity, proposing that the genotypes’ transportation among these various areas had occurred. According to our information, no research has used *S. Enteritidis* and *S. Kentucky* isolates from various origins such as calf and poultry for an ERIC PCR analysis. The goal of the current investigation was to evaluate how well the ERIC PCR could be used to analyze the genetic link between two strains. To our knowledge, there are no pervious data reported in Egypt at this point in the research. These results proved that infection by *Salmonella* may be acquired through contaminated water which was used for drinking or via contaminated food from wild birds, as reported by [15] about water, feed, and dust as a source for *Salmonella* infection. This is result is similar to the result of the authors of [53], who reported that poultry isolates had more similarities with cattle isolates than human isolates by the ERIC PCR.

## 5. Conclusions

Salmonella isolates that produced both MDR and ESBL frequently included class 1 integrons. The high incidence of class 1 integrons provides evidence that antimicrobial gene cassettes mediated by integrons play a significant role in the Salmonella resistance profile. According to the results of the current investigation, there is a significant level of clonal heterogeneity in the strain typing of lactamase-producing MDR Salmonella obtained from distinct clinical samples. One explanation for the presence of a few clonally related groups among these isolates is either clonal transmission or the propagation of antibiotic resistance through the use of various lactamases.

## Figures and Tables

**Table 1:** Samples taken for *Salmonella* isolation.

Animal Spp.	Type of Sample	Age	Number
Calves	Rectal swabs and feces	First month	40
		1–2 month	30
		2–3 month	30
Chickens	Native breeds	One day until 45 days old	40
	Cross breeds		30
	Foreign breeds		30
Total			200

**Table 2:** Sequencing of primers, target genes, sizes of amplicons, and cycling conditions.

Target Gene	Primers Sequences	Amplified Segment (bp)	Primary Denaturation	Amplification (35 Cycles)			Final Extension	Reference
				Secondary Denaturation	Annealing	Extension		
ERIC	ATGTAAGCT CCTGGGGAT TCAC	Variable	94 °C 5 min	94 °C 30 s	52 °C 40 s	72 °C 1 min	72 °C 12 min	[21]
	AAGTAAGTG ACTGGGGTG AGCG							
Integrase gene (hep 35 and hep 36 primers)	TGCGGGTYAARGATBTKGATTT	491			55 °C 40 s	72 °C 45 s	72 °C 10 min	[22]
	CARCACATGCGTRTARAT							
class 1 integron cassettes(5’-CS and 3’-CS)	GGCATCCAA GCAGCAAG	Variable			55 °C 40 s	72 °C 45 s	72 °C 10 min	[23]
	AAGCAGACT TGACCTGA							
*BlaIMP*	CATGGTTTGGTGGTTCTTGT	488			53 °C 40 s	72 °C 45 s	72 °C 10 min	[24]
	ATAATTTGGCGGACTTTGGC							
*blaVIM*	AGTGGTGAGTATCCGACA	280			53 °C 30 s	72 °C 30 s	72 °C 10 min	
	ATGAAAGTGCGTGGAGAC							
*BlaTEM*	ATCAGCAATAAACCAGC	516			54 °C 40 s	72 °C 45 s	72 °C 10 min	[25]
	CCCCGAAGAACGTTTTC							
*blaSHV*	AGGATTGACTGCCTTTTTG	392			54 °C 40 s	72 °C 40 s	72 °C 10 min	
	ATTTGCTGATTTCGCTCG							

**Table 3:** Prevalence of *Salmonella* among the tested samples.

Animal Spp.	Type of Sample	Age	Number of Samples	Positive %
Calves	Rectal swabs and feces	First month	40	4
		1–2 month	30	3
		2–3 month	30	1
Total			**100**	**8%**
Chickens	Native	One day until 45 days old	40	1
	Cross breed		30	0
	Foreign		30	1
Total			**100**	**2%**

**Table 4:** Serological identification of *S. Enterica*.

Serotype	Number	%	O Antigen	H Antigen	
				Phase 1	Phase 11
*Salmonella Kentucky*	3	30%	8, 20	I	Z6
*Salmonella Typhimurium*	3	30%	1, 4, [26], 12	I	1, 2
*Salmonella Enteritidis*	4	40%	1, 9, 12	g, m	-

**Table 5:** The prevalence rates of isolated *Salmonella* serotypes from diseased poultry and calves.

Types of Flock	Salmonella Positive	The Isolated Serotypes			
		*Salmonella* Serotypes	Number of Serotypes	Proportion/Source	Total Proportion
Diseased poultry	8	*Salmonella Kentucky*	2	25%	20%
		*Salmonella* *Typhimurium*	3	37.50%	30%
		*Salmonella Enteritidis*	3	37.50%	30%
Diseased calves	2	*Salmonella Kentucky*	1	50%	10%
		*Salmonella Enteritidis*	1	50%	10%

**Table 6:** Incidence of antimicrobial sensitivity test (AST) in *S. Enterica*.

Antimicrobial Class	Antimicrobial Agent(s) Tested	*Salmonella* Isolates		
		Resistant No. (%)	Intermediate No. (%)	Sensitive No. (%)
Quinolones	Nalidixic acid (NAL)	9 (90)	1 (10)	0 (0)
Aminoglycosides	Streptomycin (STR)	6 (60)	4 (40)	0 (0)
	Kanamycin (KAN)	4 (40)	3 (30)	3 (30)
Cephalosporins	Ceftriaxone (CRO)	0 (0)	4 (40)	6 (60)
	Cefotaxime (CTX)	1 (10)	6 (60)	3 (30)
Penicillins	Amoxicillin (AMC)	8 (80)	2 (20)	0 (0)
	Ampicillin (AMP)	9 (90)	1 (10)	0 (0)
Phenicols	Chloramphenicol (C)	1 (10)	3 (30)	6 (60)
Macrolides	Erthyromycin (E)	10 (100)	0 (00)	0 (0)
	Vancomycin (VA)	9 (90)	1 (10)	0 (0)
Tetracycline	Tetracycline (TE)	4 (40)	4 (40)	2(20)
Fluoroquinolones	Ciprofloxacin (CIP)	1 (10)	4 (40)	5 (50)
	Enrofloxacin (ENR)	2 (20)	4 (40)	4 (40)
Sulphonamides	Trimethoprim/sulfamethoxazole (SXT)	4 (40)	5 (50)	1 (10)

**Table 7:** The Jaccard/Tanimoto coefficient and the number of intersecting elements for every 2 samples with each other.

	Number of Intersecting Elements	Jaccard/Tanimoto Coefficient
Samples 3, 4	2	1
Samples 1, 2	3	0.33
Samples 1, 3	1	0.17
Samples 1, 4	1	0.17
Samples 2, 3	1	0.13
Samples 2, 4	1	0.13

**Table 8:** Agglomeration schedule of dendrograms showing genetic relationships among *Salmonella* serovars based on ERIC PCR. The similarity was calculated from the coefficient by the UPGMA.

Stage	Cluster Combined		Coefficients	Stage Cluster First Appears		Next Stage
	Cluster 1	Cluster 2		Cluster 1	Cluster 2	
1	3	4	0.000	0	0	3
2	1	2	3.000	0	0	3
3	1	3	7.500	2	1	0

## Data Availability

The authors elect to not share the data.
